# Quantifying the Length and Variance of the Eukaryotic Cell Cycle Phases by a Stochastic Model and Dual Nucleoside Pulse Labelling

**DOI:** 10.1371/journal.pcbi.1003616

**Published:** 2014-07-24

**Authors:** Tom Serge Weber, Irene Jaehnert, Christian Schichor, Michal Or-Guil, Jorge Carneiro

**Affiliations:** 1 Instituto Gulbenkian de Ciência, Oeiras, Portugal; 2 Department of Biology, Humboldt-Universität zu Berlin, Berlin Germany and Research Center ImmunoSciences, Charité - Universitätsmedizin Berlin, Berlin, Germany; 3 Tumorbiological Laboratory, Neurosurgical Department, Ludwig-Maximilians-University Munich, Klinikum Grosshadern, Munich, Germany; Memorial Sloan-Kettering Cancer Center, United States of America

## Abstract

A fundamental property of cell populations is their growth rate as well as the time needed for cell division and its variance. The eukaryotic cell cycle progresses in an ordered sequence through the phases 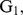 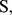 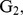 and 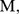 and is regulated by environmental cues and by intracellular checkpoints. Reflecting this regulatory complexity, the length of each phase varies considerably in different kinds of cells but also among genetically and morphologically indistinguishable cells. This article addresses the question of how to describe and quantify the mean and variance of the cell cycle phase lengths. A phase-resolved cell cycle model is introduced assuming that phase completion times are distributed as delayed exponential functions, capturing the observations that each realization of a cycle phase is variable in length and requires a minimal time. In this model, the total cell cycle length is distributed as a delayed hypoexponential function that closely reproduces empirical distributions. Analytic solutions are derived for the proportions of cells in each cycle phase in a population growing under balanced growth and under specific non-stationary conditions. These solutions are then adapted to describe conventional cell cycle kinetic assays based on pulse labelling with nucleoside analogs. The model fits well to data obtained with two distinct proliferating cell lines labelled with a single bromodeoxiuridine pulse. However, whereas mean lengths are precisely estimated for all phases, the respective variances remain uncertain. To overcome this limitation, a redesigned experimental protocol is derived and validated *in silico*. The novelty is the timing of two consecutive pulses with distinct nucleosides that enables accurate and precise estimation of both the mean and the variance of the length of all phases. The proposed methodology to quantify the phase length distributions gives results potentially equivalent to those obtained with modern phase-specific biosensor-based fluorescent imaging.

## Author Summary

Among the important characteristics of dividing cell populations is the time necessary for cells to complete each of the cell cycle phases, that is, to increase the cell's mass, to duplicate and repair its genome, to properly segregate its chromosomes, and to make decisions whether to continue dividing or enter a quiescent state. The cycle phase times also determine the maximal rate at which a dividing cell population can grow in size. Cell cycle phase completion times largely differ between cell types, cellular environments as well as metabolic stages, and can thus be considered as part of the phenotype of a given cell. Our article advances the methods to quantitatively characterize this phenotype. We introduce a novel phase-resolved cell cycle progression model and use it to estimate the mean and variance of the cycle phase completion times based on nucleoside analog pulse labelling experiments. This classic workhorse of cell cycle kinetic studies is revamped by our approach to potentially rival in accuracy and precision with modern phase-specific biosensor-based fluorescent imaging, while superseding the latter in its application scope.

## Introduction

The cell cycle is one of the most fundamental processes in biology. Through this process, a parental cell transmits to its two daughter cells genetic and epigenetic information by accurately replicating its DNA and evenly apportioning all nuclear and extranuclear contents. The mechanism of cell cycle regulation is tailored to ensure accurate cellular content replication, but seems to be less constrained by how long it takes to complete this process successfully. Several check points exist that ensure that chromosomes are faithfully copied and that the parental cell has enough material in order to produce two viable isogenic daughter cells. Meeting the conditions of each of these check points takes variable time and delays the completion of the cell cycle. Yet, how long the cells take on average to complete the cell cycle is an important biological property. In unicellular organisms, the average intermitotic time is a direct measurement of the organism's fitness, while in multicellular organisms, the regulation of the rate of cell division is critical for development, stem cell maintenance, tissue or organ homeostasis, wound healing, and immunity. The temporal organization of the cell cycle is therefore under tight regulation, likely reflecting a fine balance between accuracy in information transmission and speed.

The average cell cycle time has been estimated at the population level by measuring the growth curve of exponentially proliferating cell cohorts, under conditions in which cells can be counted and cell death is negligible compared to the population wide growth rate. Under conditions in which cell counting is not possible or in which cell death rates cannot be neglected (e.g., homeostasis, immune reactions, cancer growth), indirect estimates for the average division time or the average death time are typically inferred e.g., through the rate of increase of cells arrested in mitosis after administration of colchicine, the fraction of labelled mitotic figures after pulse labelling (FLM method), and from long-term labelling and delabelling time-series of deuterium or bromodeoxyuridine (BrdU) tracing experiments [1]–[3]. For growing cell populations these estimates depend on assumptions about the shape of the intermitotic time distribution [4]. The latter, when analyzed at a single-cell level, e.g., by time-lapse imaging, shows significant variability in otherwise seemingly homogeneous cell populations. This observation led more than forty years ago to the development of one of the first stochastic cell cycle models [5]. Smith and Martin proposed at that time that cell's life comprehends an 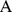 state and a 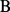 phase. Whereas the time cells spend in the 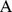 state was assumed to be exponentially distributed, the time cells spend in the 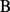 phase was, in this simplest scenario, a fixed delay. Experimental validation was provided by time-lapse imaging of growing cell cultures, measurements of fraction of labelled mitoses and fractions of sibling pairs with age difference greater than a specified value [6]. Even though later studies [7]–[10] have shown that the model assumptions do not exactly match experimental data, its simplicity and mathematical tractability makes the Smith-Martin model even today a popular theoretical model [6], [11].

In the last ten years, 5-(and 6)-Carboxyfluorescein diacetate succinimidyl ester (CFSE) dilution assays in concert with a whole set of advanced modeling techniques [12]–[14] allowed to estimate the average duration, as well as inter-cellular variability in more complex scenarios with division time densities *in vitro* or *in vivo* after adoptive cell transfer. Especially generation structure, activation times and generation dependent cell death were included in these models and subsequently estimated in the context of lymphocyte proliferation. Inter-cellular variability not only of division times but also of death times were confirmed directly in long-term tracking of single HeLa cells [15] and B-lymphocytes [10]. The latter study provided extensive quantitative data on the shape of age-dependent division and death time distributions which are required to calibrate e.g., the Cyton [16] or similar models. A review on these, and alternative stochastic cell cycle models is given in [4].

At a higher temporal and functional resolution the eukaryotic cell cycle is structured into four distinct phases: 1) the 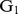 phase during which organelles are reorganized and chromatin is licensed for replication, 2) the 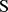 phase in which the chromosomes are duplicated by DNA replication, 3) the 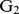 phase which serves as a holding time for synthesis and accumulation of proteins needed in 4) the 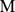 phase, or mitosis, which is marked by chromatin condensation, nuclear envelope breakdown, chromosomal segregation, and finally cytokinesis, which completes the generation of two daughter cells in 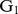 phase [17].

Considering explicitly cell cycle phases in mathematical models of cell division probably dates back to the discovery that 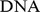 is replicated mainly during a specific period of the cell cycle. Already in their seminal paper, Smith and Martin related the 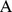 state to the 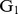 phase and the 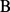 phase to the 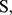 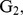 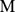 and possibly to some part of the 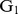 phase. Subsequent studies that explored phase-resolved cell cycle models, majoritarely rooted in the field of oncology and cancer therapy, include [18]–[25]. As in the present work, most of these studies relied on flow cytometry 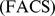 data generated by labelling selectively cells that are synthesizing 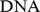 using nucleoside analogs (e.g., BrdU, iodo-deoxyuridine (IdU) or ethynyl-deoxyuridine (EdU)), together with a fluorescent intercalating agent to measure total DNA content (e.g., 4,6- diamidino-2-phenylindole (DAPI), and propidium iodide (PI)), in order to test the model assumptions and draw conclusions about the cells and conditions under consideration.

Here we present a simple stochastic cell cycle model that incorporates temporal variability at the level of individual cell cycle phases. More precisely, we extend the concept underlying the Smith-Martin model of delayed exponential waiting times to the cell cycle phases. We first demonstrate that the model is in good agreement with published experimental data on inter-mitotic division time distributions. We then show, based on stability analysis, that phase-specific variability remains largely undetermined when measurements are taken on cell populations under balanced growth (i.e., growth under asymptotic conditions in which the expected proportions of cells in each phase of the cycle are constant). We prove that by properly measuring proliferating cells under unbalanced growth, one can with at least three well placed support points, assuming noise-free conditions, uniquely identify the average and variance in the completion time of each of the cell cycle phases. When comparing our model with two experimental data sets obtained from conventional pulse-labelling experiments of distinct proliferating cell lines, we find that, while the kinetics extracted from these experiments are well approximated by the predictions of the proposed model, the information content is insufficient to determine accurately all the parameters. Finally we propose a modification of the prevailing experimental protocol, based on dual-pulse labelling with 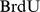 and, for example, 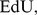 that overcomes this shortcoming.

## Results

### Model definition

**Figure 1:**
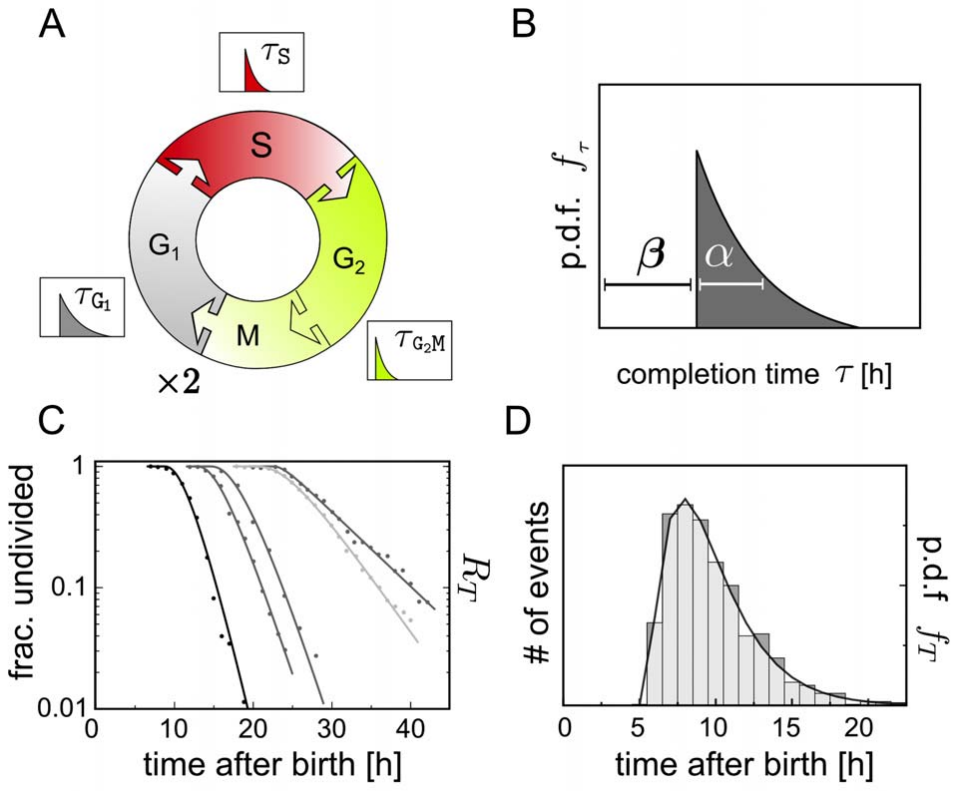
Stochastic cell cycle model. **A**: Scheme of the proposed cell cycle model with three phases 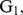 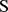 and 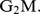 The dashed border between the 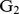 and the 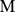 phase indicates that the 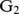 and 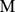 phase are pooled into a single phase. The random time 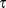 a cell needs to complete the processes associated to each of the phases, follows a delayed exponential distribution with specific parameters 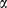 and 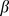 for each phase. **B**: Delayed-exponential completion time distribution density 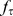 with parameters 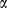 and 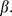 **C**: Best fit of the complementary cumulative distribution 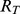 to the fraction of undivided cells after birth obtained by time lapse cinematography [5] of slow and fast dividing cell lines. **D**: Best fit of 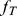 defined by Eq. 4 (solid line) to inter-mitotic time distribution density measured by long-term video tracking of *in vitro* proliferating B-cells [10]. The data in C and D were read from the graphs in the original publications ([5] and [10] respectively).



The eukaryotic cell cycle is defined as an orderly sequence of three phases distinguished by cellular DNA content, termed 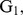 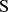 and 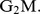 A dividing cell is supposed to proceed, under this minimalist view, from one phase to another in a fixed order, until reaching the end of 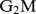 phase. Here it completes cytokinesis generating two genetically identical daughter cells that are by definition in 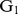 phase (Fig. 1 A). We assume that the completion time of any phase (i.e. the time lapse between the entry to and exit from that given phase) is a random variable 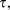 which is distributed according to a delayed (or shifted) exponential density function (Fig. 1 B),

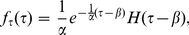
(1)where 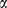 is the reciprocal of the rate of the exponential (measured in 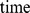 units) and 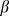 is the fixed delay (in 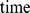 units), and 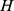 denotes the Heaviside step function whose value is zero for negative argument, i.e., for 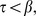 and one for positive argument. Notice that with a slight abuse of notation we denote here the random variable (subscript of density function 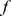) and the value it assumes (the argument of the function 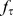) by the same symbol 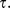 This will allow us to denote the probability density function and the cumulative probability distribution of the random variable 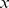 by 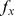 and 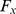 respectively, and to define the complementary cumulative distribution 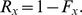 The delay 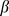 in Eq. 1 ‘ensures’ that a cell that enters a specific phase will remain therein for at least 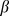 time units (e.g. hours) before proceeding to the next phase. Besides this fixed minimal time 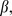 additional less predictable effects that affect the completion of the processes associated to a phase are assumed to be exponentially distributed with both mean and standard deviation given by 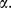 The phase specific mean completion time, denoted in the following by 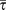 is then 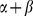 with standard deviation 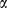 and coefficient of variation 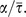 The Laplace transform of Eq. 1 is given by
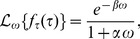
(2)


where 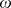 is the transformed variable corresponding to the time lapse 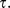 The temporal organization of the cell cycle is defined by the vector of phase-specific completion times, 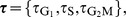 which in turn depend on the parameter vectors 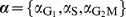 and 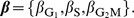 The cell cycle length, understood as the time lapse between the entry into 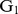 until exit out of 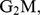 is the random variable 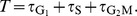 Its probability density function is the convolution of the three underlying delayed exponential distributions and corresponds to the delayed hypoexponential distribution. Explicit expressions can be computed using the inverse Laplace transform 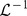 of the product of the Laplace transforms of the three densities given by Eq. 2, i.e.,
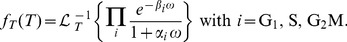
(3)


In case that all entries in 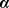 are distinct, we get
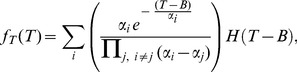
(4)


in which the indices 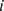 and 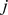 iterate over the three phases and 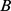 is the sum of the elements in 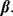


In Fig. 1 B we plot the shape of the phase specific completion time distribution 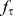 defined by Eq. 1, which illustrates that the probability for a cell to complete a given phase in less than 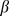 time units is zero under this model. A graphical representation of the cell cycle model is provided in Fig. 1 A. Notice that each phase can have distinct parameter values 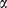 and 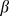 for the completion time distribution.

As a first validation, we compared the empirical frequency of undivided cells as a function of time after ‘birth’ (reported by [5]) with the respective probability according to the model 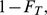 which we denote as 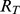 (Fig. 1 C). As a second test, we fitted the cell cycle length density 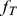 given by Eq. 4 to data extracted from video-tracking of *in vitro* proliferating B cells [10]. The delayed hypoexponential distribution 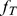 (shown in Fig. 1 D), but also the delayed log-normal and the delayed gamma distribution (not shown) with parameter values proposed in [10], reproduce closely the measured division time histogram. While the two latter depend on three parameters each, the hypoexponential distribution depends on six parameters, that remain largely undetermined given this kind of data.

### Balanced growth

A proliferating cell population that obeys the probability model specified in the previous section can be represented by a non-Markov multidimensional random process, whose evolution depends on its history. There exist an infinite number of possible histories or realizations of the population size dynamics 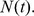 We focus here on a specific important subset, namely those under balanced growth. Under balanced growth a cell population grows exponentially 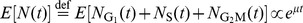 with mean growth rate 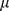 and constant mean proportions of cells in the three phases 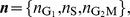 where e.g., 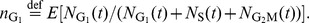 The expectation operator 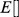 is defined over all possible realizations of the process.

We will now derive explicit expressions for 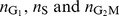 and a transcendental equation that defines 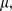 the growth rate. A first step in obtaining the constant frequencies of cells in each of the phases consist in computing the ratio between the cells that complete a given phase and the total number of cells inside the same phase at time 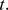 This phase-specific quantity, denoted here by 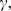 represents the asymptotic efflux rate constant, which will be useful, as we will see, to construct a transition probability matrix 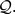 The latter will enable us to employ methods from linear algebra to solve the steady state condition.

Suppose for example that a cohort of cells entered a given phase at time 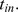 Then the density of cells leaving this phase at time 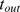 will be 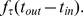 Similarly if a cohort of cells entered this phase at time 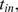 then a proportion 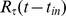 will remain in it until time 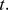


Recalling that the influx of cells into a given phase is proportional to 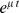 and that 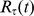 is the complementary cumulative distribution of 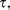 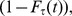 which is Laplace transformed to 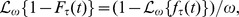 we integrate over all past entries and finally take the ratio to obtain
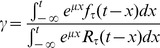
(5)

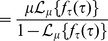
(6)

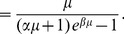
(7)


While the second equality is a consequence of the definition of the Laplace transform, the third equality follows by substituting 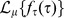 using Eq. 2. For a phase without a delay, i.e., 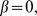 the last expression simplifies to the familiar mass action principle, where the transition probability is directly proportional to the decay rate 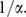 Assuming that cells are immortal and recalling that division occurs as cells proceed from 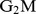 to 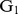 we build up the transition probability matrix as follows
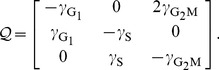
(8)


The balanced growth condition can now be formulated in matrix form
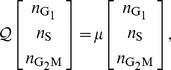
(9)


where the growth rate 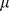 is an eigenvalue of 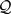 and the proportions vector 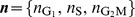 is the corresponding eigenvector. It can be shown that there exists a single dominating real positive eigenvalue for 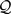 (see Materials and Methods) whose associated normalized eigenvector is
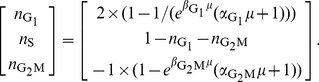
(10)


The uniqueness and existence of a dominating positive real root ultimately motivates our focus on balanced exponential growth, as any immortal proliferating cell population with sufficient nutrients and space will eventually enter this stationary phase. The time it takes, either starting with a single cell or a synchronized cell cohort to enter this state depends on the cell cycle parameters. The exponential growth rate 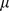 is the unique real positive root of the characteristic equation 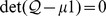 which writes as
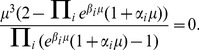
(11)


It is easy to see that the denominator in Eq. 11 is always positive. To determine a non-trivial 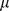 it remains to solve the transcendental equation in the numerator
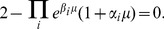
(12)


Numerical solutions to this equation can be computed using e.g., the Newton-Raphson root finding algorithm, with fast convergence if the initial value is set to 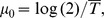 where 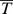 is the average cell cycle length, i.e., the sum of the elements in 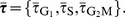 This first guess is a naive estimate for 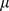 assuming that cells divide according to a deterministic division time identical to the average of the hypoexponential density defined in Eq. 3.

### Learning from cell frequencies measured under balanced growth

The predicted fractions of cells in each of the phases can be compared to frequencies extracted experimentally from bivariate analysis of cell populations transiently exposed to nucleoside analogs and subsequently examined both for the intensities of the signals due to incorporated nucleoside analog and total DNA content [26] (e.g. the so called BrdU-DAPI staining dot plot). The question that we want to address in this section is: What can potentially be learned about the parameters of the model, given this type of experimental data? By definition, the measured frequencies will sum to one, and therefore we have for three populations effectively only two equations but six model parameters. This makes it impossible to identify all the parameter values, irrespective of the number of samples we take. It is however possible to derive analytical expressions for the upper and lower bounds for both the parameters and the average completion time of each phase.

Consider the experimentally determined frequencies, denoted by 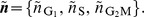 Substituting the vector 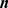 by 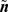 in Eq. 10 and solving for each phase specific parameter 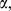 we obtain
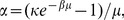
(13)


where 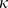 is a phase specific element of the vector
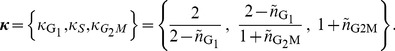
(14)


The phase specific parameters 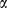 and 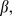 respectively the reciprocal rate and delay, are by definition greater or equal to zero. These conditions propagate into Eq. 13 which allows us to specify boundaries for 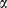 and 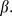 First notice that 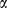 is, for each phase, a monotonically decreasing function of 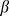 with a maximum 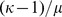 at 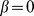 and a zero crossing at 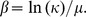 The maximum and the root represent the upper bounds for 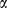 and 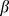 respectively, while the lower bounds are zero for both. We thus have for each phase
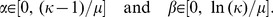
(15)


The mean phase-specific completion time, 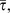 the sum of the reciprocal rate 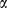 and the delay 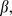 is also bounded, with an interval given by
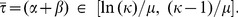
(16)


This result is derived from the fact that 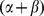 is concave having its unique minimum at 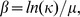 which follows from setting the derivative 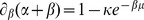 to zero. This implies that 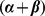 is a monotonically decreasing function in the interval 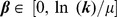 with the corresponding extrema specified above. It is important to note that the intervals defined by Eqs 13–16 depend on the average growth rate 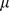 which is in general not known. Formally if one specific pair of parameter vectors 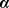 and 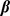 explains the measured frequencies with growth rate 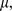 the scaled parameter vectors 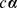 and 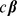 mimic equally well the same data for arbitrary positive 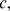 however with a reduced growth rate 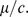 This can be easily verified by substituting these expressions in Eq. 10 and Eq. 12. The direct consequence is that 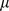 remains undefined. However for the relative average time a cells spends e.g., in 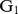 phase 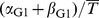 the growth rate cancels out.

Using the fact that 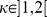 and the appropriate series expansion for the natural logarithm, the widths of the intervals bounding 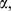 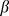 and 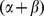 for each phase can be written as:
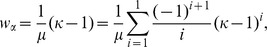


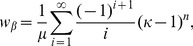
(17)

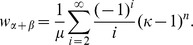



From this it is straight-forward to show that 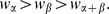 This implies that by using measurements of the phase-specific stationary cell frequencies to infer the phase-specific completion times 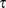 results in estimates of the mean value 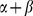 that are more precise than the estimates of the standard deviation 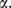 Notice that the width of the intervals can be interpreted as a naive lower bound for the uncertainty about the respective parameter values. For the two data sets analyzed in this article (see details in next section), we computed the intervals for the phase-specific standard deviations 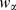 that were on average 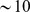 times wider than the intervals for the expected phase-specific completion times 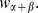


### Transient unbalanced growth

Balanced growth analysis does not allow to distinguish between fixed (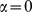) and purely exponentially distributed (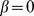) completion times even if 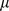 is known. This follows from Eq. 15 because possible values for the standard deviation 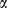 include 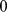 and 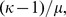 and the latter requires, according to Eq. 16, the delay 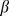 to be null.

The incapacity to resolve the values of 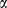 and 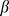 is overcome if one selects and follows a subpopulation within which the proportions of cells in each phase are transiently different from the balanced growth proportions. Consider a simple *thought* experiment that consists in taking a population under balanced growth and labelling all the cells that are in a specific phase, say 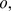 which can be either 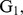 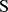 or 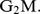 Initially all the cells are in the same phase 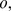 but as time passes by the labelled cells progress through the cell cycle and eventually distribute over the three phases. The labelled cell subpopulation which is initially not balanced will return asymptotically to balanced growth conditions, restoring the corresponding proportions of cells in the three phases. We refer to this transient dynamics of a selected subpopulation as transient unbalanced growth. It turns out that measuring the transient dynamics of this subpopulation yields information that potentially allows to distinguish between a fixed and a purely exponentially distributed phase completion time. More specifically, a mathematical proof will show that taking samples at three well chosen time points (support points) permits under ideal conditions accurate estimation of the average and the variability in the time required to complete the phase 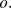


The initial average fraction of cells in phase 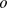 which are selectively labelled at time 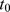 is determined by Eq. 10. To predict when the labelled cells will have completed 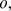 we need to specify when they entered this phase. For the time before labelling the average influx into 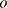 is proportional to 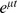. For the time after the labelling, because by definition all labelled cells entered phase 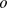 before 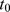 (otherwise they would not be labelled ‘as being in phase 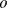
’), the entry of cells is zero. Hence, the average influx to the labelled subpopulation is proportional to 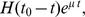 where 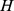 denotes the Heaviside step function. Let us assume that within the subpopulation of labelled cells and their progeny one could identify how many phases a cell or a cohort of cells went through since the labelling event, and let 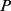 count the number of phases since labelling.

In close analogy to expression Eq. 5 we compute the time-dependent exit-rate density distribution for cells with 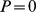 as
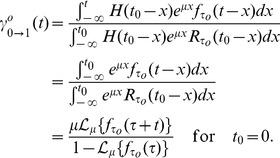
(18)


where, for convenience, we interpreted and will interpret in the following 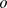 both as a phase and a phase index. As before, the third row follows from the definition of the Laplace transform setting 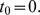. On the left-hand side, the arrow from 0 to 1 represents the transition from the initial phase 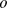 (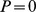) to the next phase (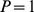), corresponding to the completion of the initial phase 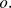 In contrast to Eq. 5, the denominator accounts for the cells that entered or initiated phase 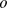 sometime in the past, and did not complete this phase until the instant of labelling 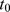 (and not at time 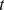 as in Eq. 5), while the numerator, except for the altered average influx, remains unchanged.

After computing 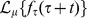 and substituting 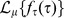 using Eq. 2, Eq. 18 yields for 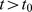


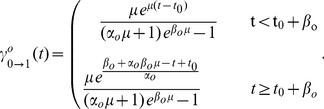
(19)


It follows that the accumulated average cell flux that at time 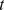 has completed 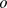 and progressed to the next phase is given by
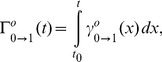
(20)


which for 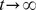 approaches one, reflecting the fact that all cells will eventually complete 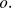


The Laplace transform of Eq. 20 writes as
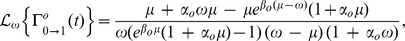



where 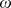 is, as before, the transformed variable corresponding to 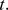


Within a cohort of cells isolated for instance in 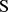 phase, i.e., 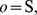 the accumulated average cell flux out of the subsequent 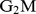 phase can then be derived recalling Eq. 2 and using the properties of the inverse Laplace transform as
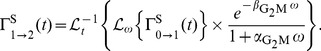
(21)


For an arbitrary cell cohort originally in 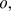 the accumulated average flux, completing 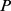 phases and entering the 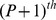 phase since isolation, can be written in general as
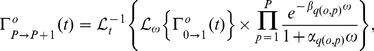
(22)


in which 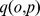 denotes a function which returns an appropriate phase index. For 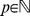 and 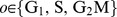 it is defined as
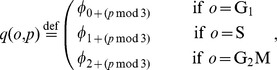



where 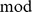 is the modulo operation, and 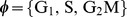 is a vector of cell cycle phase indices. The function 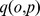 thus returns, for increasing 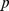, in a cyclical fashion, the cell cycle phase indices, starting with 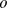 for 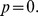 Notice that Eq. 21 corresponds to Eq. 22 for 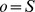 and 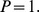


Analytical expression for Eq. 22, although solved relatively easily with modern algebra software, can become quite cumbersome for values of 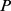 larger than six. In our case, deriving the expressions for 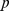 up to a value of five was sufficient to simulate the experiments.

Because we want to compare the model predictions with experimentally measured cell frequencies, more interesting than the accumulated fluxes are the expected proportions of cells inside each phase over time. These can be computed using Eqs 20–22, closely following the methodology outlined in [11], [12]. For the fraction of cells initially in phase 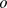 we have
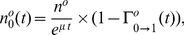
(23)


where the lower index 0 in 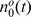 indicates that this expression describes cells which completed zero phases since 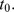 The first term on the right hand side corresponds to the fraction of cells in phase 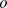 at 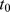 divided by 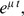 which accounts for the total population growth during the same interval. The second term stands for the fraction of cells that remained in phase 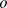 up to time 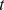 relative to the initial number of cells in this phase. By evaluating the integral in Eq. 20, substituting in Eq. 23 and letting as before, without loss of generality, the time of partition 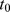 be zero, we get for 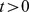


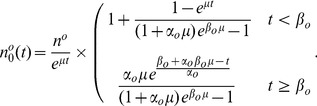
(24)


Expressions for cells initially in 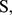 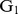 or 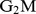 phase can be obtained by substituting 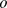 by the respective phase.

If there were no cell division (i.e., 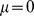) we could readily obtain the average fraction of cells that completed 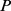 phases at time 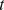 as the difference between the cells that entered the 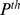 phase, i.e., 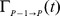, and those that left it, i.e., 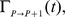 divided by 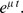 To account for cell division, we need to multiply this difference by an additional term 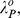 which increases by a factor 2 each time cell cohorts make a transition from 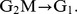 This term is defined, for each case, as follows: 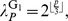 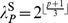 and 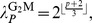 where the brackets in the exponent represent the floor operator.

In general we get for all consecutive phases for cells initially in phase 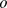 the relatively manageable expression
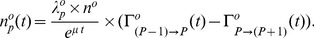
(25)


As for Eq. 24, the resulting solutions are defined as piecewise-continuous functions in time. Also notice that most expressions in this section can be written in more compact, but less intuitive, vector form, by dropping the initial phase index 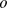 and using bold vector notation as before.

### Learning from cell frequencies measured in transiently unbalanced growing subpopulations

In this section we will show that data from the transient kinetics generated by our *thought* experiment allows to accurately estimate the average and the variability in the individual completion times. The proof is based on the analytical expressions derived in the previous section, and also on the assumption that the kinetics are acquired under the ideal conditions of large population sizes and no measurement errors. The latter condition, although clearly unrealistic, can always be approached in practice by increasing the number of samples at each support point.

For the sake of generality, consider a subpopulation of cells that are in an arbitrary phase and are labelled at 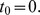 Assuming that the ‘label’ does not in any way affect the cell cycle of the cells, the parameters 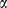 and 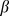 of the labelled subpopulation are the same as those of the full population under balanced growth. Under these conditions, we can obtain 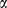 using Eq. 13 and Eq. 14 with the fractions 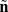 of the full population observed at time 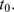 Substituting 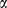 in the upper row of Eq. 24 and solving for 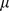 to find
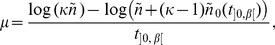
(26)


where 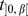 denotes an arbitrary time point that lies in the interval 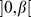, 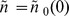 and 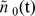 is the experimentally determined equivalent of Eq. 24. This shows that the balanced growth rate 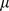 is fully determined by only two support points, one immediately after the partition at 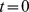 and a second at an arbitrary 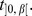 This also makes clear that placing more support points in the interval 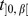 does not increase knowledge about 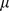 nor the parameter values, under ideal conditions. Importantly the uncertainty about the phase-specific variability discussed in previous sections remains.

By replacing the same expression for 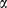 in the second row of the right-hand side of Eq. 24 we get
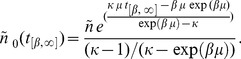
(27)


After experimentally acquiring 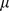 and the phase specific 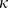 and 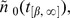 this expression will depend on a single unknown 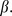 One can show that Eq. 27 is solved by a unique 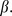 This follows from the fact that the right hand side of Eq. 27 is a monotonically decreasing function in 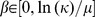 with corresponding values lying in the interval 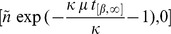 while the left hand side is positive by definition. Substituting the solution for 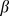 into Eq. 13 yields the remaining parameter vector 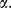


Taken together this proves that in theory samples of the three cell cohorts 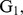 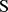 and 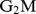 taken at three support points, a first at 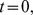 a second at 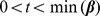 and a third at 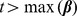 are sufficient to determine all the parameters of the model.

### Conventional single pulse-labelling assays

The *thought* experiment analyzed so far, although conceptually simple, poses a series of experimental challenges, that make a one-to-one realization difficult. The technical difficulties lie mostly in initially separating the cells according to their phase and in following these cells as they enter the subsequent phases. A widely used technique, namely DNA-nucleoside-analog pulse-chase labelling experiments, generates nevertheless to a certain extent comparable data. The latter achieves the initial phase-specific partitioning by exposing during a short time window proliferating cells with a nucleoside analog (e.g., BrdU, IdU or EdU) that gets selectively incorporated into the DNA of cells that are actively replicating their genome. Measuring subsequently by 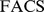 simultaneously the DNA content and the amount of incorporated nucleoside analog per cell permits to discern the three phases 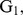 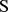 and 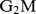 immediately after the pulse. In addition, due to the permanent staining property of the nucleoside analogs, it is possible to follow, up to a certain degree, the labelled and unlabelled cell cohorts over time. Several dies, such as Hoechst 33342, the dihydroanthraquinone analog DRAQ5, DAPI, and PI are commonly available to stain DNA content in cells [27], and can be used in combination with nucleotide analogs.

**Figure 2:**
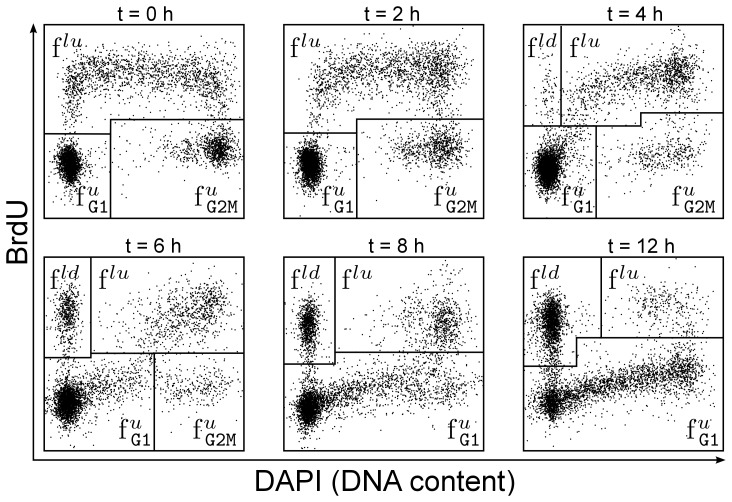
DAPI-BrdU pulse-chase labelling FACS data. Samples taken at several time points after pulse labelling proliferating U87 human glioblastoma cells with 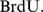 The four gated populations are 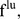 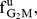 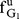 and 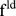 which are defined precisely in the main text. Briefly, the subscript indicates the phase at the instant of labelling, while the superscripts ‘u’, ‘lu’ and ‘ld’ refers to cells ‘unlabelled’, ‘labelled and undivided’ and ‘labelled and divided’, respectively. The data was generated as described in the Experimental Methods section.

In theory, this method would largely correspond to the hypothetical experiment that we analyzed so far. In practice however, the overlap of the subpopulations in the 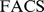 scatter plots prevents the exact determination of the frequencies of cells described by Eq. 24 and Eq. 25. For example labelled cells that have completed the 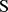 phase but remain in 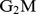 phase are indistinguishable from those that did not complete the initial 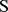 phase yet. As has been reported previously, only four different sub-populations can be identified with reasonable accuracy [26]. These are:

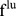
: labelled undivided cells which at time of labelling (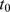) were in 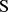 phase (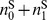)

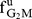
: unlabelled cells that were in 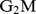 phase at 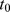 (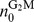)

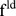
: first generation progeny of labelled cells which were initially in 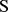 phase (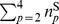)

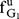
: unlabelled cells and progeny of cells that were in 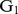 at 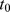 accompanied by the progeny of 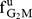 and 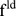 (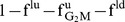)

where the corresponding populations in our *thought* experiment are indicated in brackets. This shows that computing Eq. 25 up to 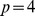 is sufficient to describe a complete *in silico* BrdU pulse labelling experiment. The reason is that, using current protocols, fluorescence of labelled cells becomes indistinguishable from background as soon as the cells divide a second time. In other words, cells that leave population 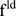 by dividing a second time join population 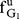 (see Fig. 2). For the experimental data, analyzed in the next section, the fraction of labelled cells that completed two cell divisions during the 12 hours time frame of the experiment is negligible.

The population 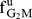 is the only sub-population that matches directly the type of data considered before and its temporal evolution follows as such Eq. 24. The remaining three populations in contrast represent mixtures of cell cohorts whose kinetics could be described individually by Eqs 24–25.

### Learning from single pulse-labelling data

By analyzing two data sets from samples of 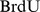 single pulse-labelling experiments, we tested the model and the effect of population intermixing on the identification of the model parameter values. The two cell lines considered were *in vitro* cultured 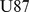 human glioblastoma cancer cells (for details see Materials and Methods) and *in vitro* cultured 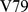 Chinese hamster cells (courtesy G. Wilson). We will refer to these data as the 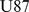 and the 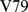 data sets. Both data sets consist of samples taken from asynchronously dividing cell populations at several time points after a single BrdU pulse, with sample sizes ranging from 5000 to 50000 cells each. Data points represent simultaneous measurements of BrdU as well as DAPI or PI (DNA content) in a single cell by fluorescent activated cell sorting.

**Figure 3:**
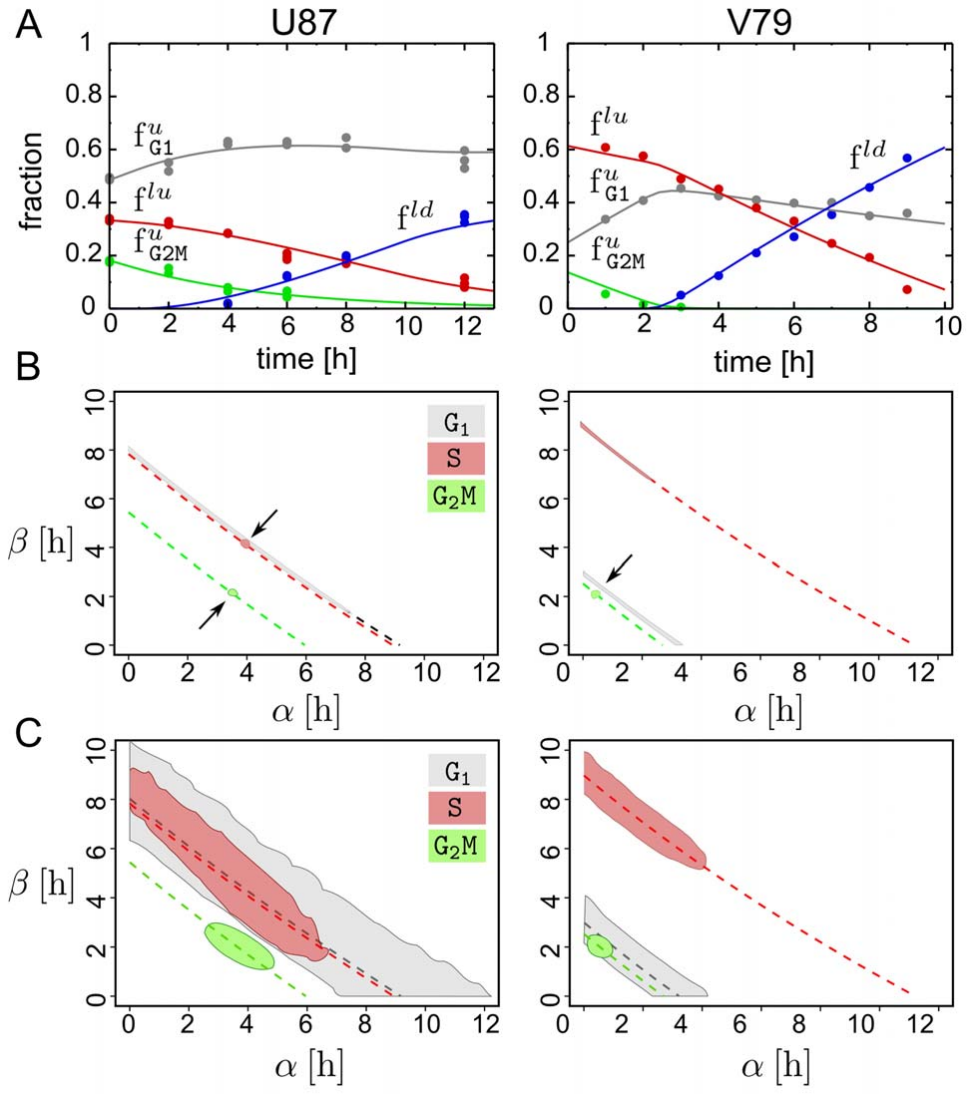
Model based parameter estimation. **A**: Best fit of the model predictions (lines) to experimentally determined cell fractions after BrdU pulse labelling (dots). U87: In vitro cultured U87 human glioblastoma cancer cell line (three replicates). V79: In vitro cultured V79 Chinese hamster cells (single replicate) (courtesy G. Wilson). Best fit parameter values used to compute model predictions (U87: 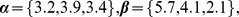 V79: 
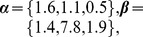
 units are hours). **B**: Approximate ML regions for the parameters 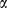 and 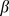 associated to each phase (gray: 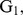 red: 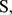 green: 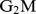). **C**: Bayesian bi-variate 99%-credibility regions for the parameters 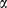 and 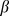 for each phase. Arrows indicate point estimates and the dashed lines delineate the information that could have been gained in our *thought* experiment under noise-free conditions from two support points, one at 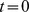 and a second at 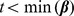. The U87 data set was generated as described in the Experimental Methods section. The V79 data set was a kind gift of G. Wilson.

As a preliminary test we minimized the residual sum of squares 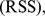 i.e., least-squares fitting, of adequate mixtures of Eq. 24 and Eq. 25 to extracted frequencies at different time points after the pulse. We found that, for properly chosen parameter values, both data sets were reasonably well approximated by the model predictions (Fig. 3 A).

While this indicated that the model captured some of the relevant temporal characteristics of cell cycle progression, a subsequent analysis revealed that an infinite number of different parameter combinations fitted the measured frequencies with the same minimal 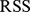 (not shown). This implies that there exist, given the available data, no single best-fit parameter combination, but a whole region in parameter space that can explain the data equally well.

When we then interrogated the same data by approximate maximum likelihood (ML) estimation, using a simple 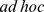 likelihood function (see Materials and Methods), we found again that relative large regions in parameter space mapped to the same ML (see Fig. 3 B). It turned out that these regions were entirely superimposed onto the lines defined by Eq. 13 and Eq. 26 (dashed lines). These lines define what could have potentially been learned in our *thought* experiment with only two support points, one at 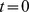 and a second at 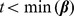. In both experiments, ML parameters associated with the 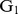 phase were spread out almost everywhere along these lines (Fig. 3 B, gray regions). Parameters related to the 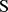 phase were more concentrated but still in the case of the 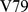 data a substantial region of ML estimates were observed. Finally the region for the 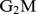 phase parameters approached that of a point estimate for both data sets.

The spread of the ML estimates suggests that even in the ideal case of large population size and noise-free data, the specific choice of the support points in these experiments does not allow to determine uniquely neither the delay nor the standard deviation for all the phases. In contrast the average completion time for each phase and the total division time can be estimated with relatively high precision.

To better quantify the uncertainty of these estimates, Bayesian 99% credibility regions (CR) were computed by the Markov chain Monte Carlo method (MCMC) using the same likelihood function as before (Fig 3 C). CRs followed mainly the same trends as the regions observed in the ML estimates, covered however as expected a larger volume. An exception was the ‘blown up’ CR of the 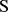 phase parameter for the 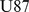 cell line, for which the ML estimates wrongly insinuated a well defined point estimate.

In Table 1 we summarized the obtained Bayesian summary statistics. One can see that the intervals for the average duration of each phase 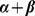 are narrow compared to those for the individual parameters 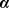 and 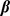. In both cases the data allows for a deterministic 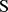 phase (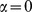), while for the 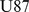 data set variability in 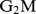 is a necessary characteristic to reproduce accurately the data. Notably, when contrasting the two cell lines, are the short 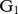 phase of Chinese hamster cells and the approximately two times more extended 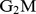 phase of the human glioblastoma cell line. It is out of the scope of this paper to interpret or relate these differences to cell line specific conditions. More importantly in this context is the fact that the information of the analyzed data is too sparse to narrow down all the parameter values even under noise-free conditions.

**Table 1:** Bayesian summary statistics.

	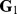			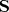			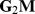			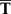
	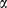	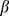	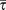	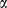	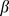	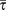	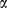	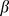	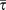	
U87	4.8	4.1	8.9	3.3	4.8	8.2	3.6	2.0	5.6	22.9
	0.0∶11.2	0.0∶9.7	6.7∶11.7	0.0∶5.9	2.0∶8.8	7.1∶9.5	2.7∶4.6	1.3∶2.7	5.1∶6.2	19.4∶26.4
V79	1.6	1.5	3.1	1.3	7.6	9.0	0.5	2.0	2.5	14.7
	0.0∶3.6	0.0∶3.4	2.5∶3.8	0.3∶3.6	5.5∶9.6	8.4∶9.7	0.2∶0.7	1.7∶2.3	2.3∶2.7	13.7∶15.9

Bayesian summary statistics (mean, 99%-credibility intervals) for individual cell cycle parameters, average durations 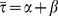 and the total cell cycle length 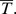 The intervals for the average durations are narrow compared to those for the individual parameters 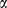 and 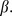 All values are given in hours.

### Redesigned dual pulse-labelling assay

The information extracted from the 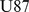 and 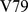 data sets is apparently insufficient to pinpoint all six parameters related to the three phases of our simple cell cycle model. This is disappointing especially because the number of support points largely exceeds the three ideally required, and the support points seem to include at least for the U87 data set one at 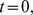 a second at 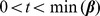 and a third at 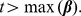


A potential explanation for this poor resolution in the estimates is the previously mentioned intermixing of the cell population clusters in the BrdU versus DAPI scatter plots compared to the ideal conditions discussed earlier. The cluster overlap in the data makes it impossible to measure directly the frequencies of most of the populations, including the cell cohorts described by Eq. 24.

**Figure 4:**
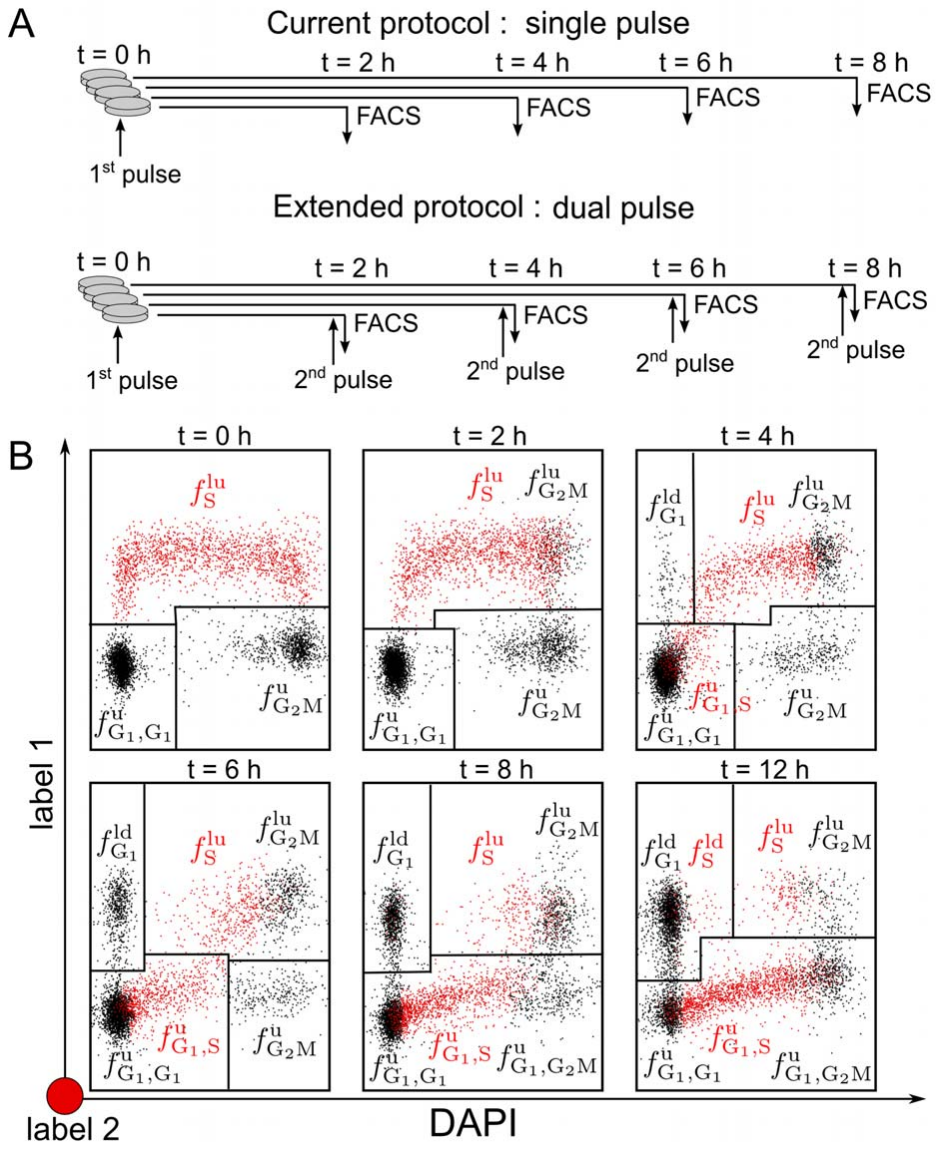
Dual pulse protocol. **A**: Simplified schematic representations of the protocols corresponding to a conventional single pulse labelling with one nucleoside analog (e.g., BrdU) and a dual pulse labelling experiment with two different nucleoside analogs (e.g., BrdU together IdU or EdU). **B**: Artificial staining of single-pulse labelling data (for original data see Fig. 2), showing eight of the nine subpopulations that could potentially be identified with double-pulse labelling. Notice that the four population 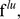 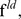 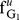 and 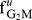 that can be followed by the conventional protocol, have each been subdivided according to the cell cycle phases. The naming convention for the populations is as follows: the superscript (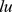 = ‘labelled undivided’, 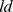 = ‘labelled divided’, 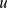 = ‘unlabelled’) indicates whether the population is labelled and whether it has divided since the time of the first pulse; the first and the second subscript (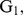 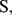 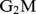) stand for the phase in which the population was at the time of the first and the second pulse respectively. Double subscripts are used only when necessary.

In order to approach the conditions assumed in the *thought* experiment by avoiding the loss of information caused by the intermixing, we devised an extension of the current single pulse protocol, which places a second pulse immediately before measuring or fixing each sample (see Fig. 4, top). The second pulse is expected to expose the cells with a further nucleoside analog that can be distinguished from the first one by 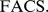 Depending on the cell cycle kinetics and the length of the measuring period, the additional pulse increases the number of classifiable populations from four up to nine distinct populations.

To appreciate the additional populations identified by double pulse labelling, data from a single pulse-chase labelling experiment was artificially colored, to mimic the expected FACS output from proliferating cells labelled according to the protocol described before. In Fig. 4, besides the gates defining the populations 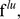 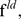 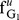 and 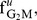 cells that have incorporated the second label are drawn in red. For the time immediately after the pulse (i.e., 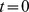), no extra information is gained by the second pulse. However, already two hours later, one additional population can be discerned. Twelve hours after the first pulse, seven population, instead of three, can be recognized. Thus by resolving the four initial population according to the cell cycle phases, it is possible to measure the kinetics of nine subpopulations (
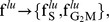
 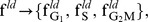 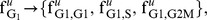 and 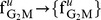). Because all these kinetics depend on the cell cycle parameters, each of them can in principle tell us something about the phase completions times. However some information is redundant. For example if 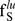 and 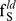 are measured, then 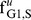 is defined by the total fraction of cells in 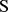 phase, because 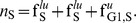 Similarly from 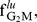 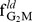 one can deduce 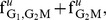 by knowing the frequency of cells in 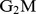 phase.

Double-label experiments using pairs of nucleoside analogs like BrdU, IdU and EdU, also in combination with radioactive tritiated thymidine (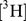), have been explored in several cancer cell proliferation studies [19], [28]–[31]. In recent years, dual pulse experiments using BrdU in combination with EdU have become more common. Studies relying on this method estimated changes in 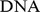 replication, inferred mitochondrial DNA bio-genesis and stained proliferating cells in the bone marrow in vivo [32]–[34], in general with the aim to increase the statistical power of the conventional methods.

**Figure 5:**
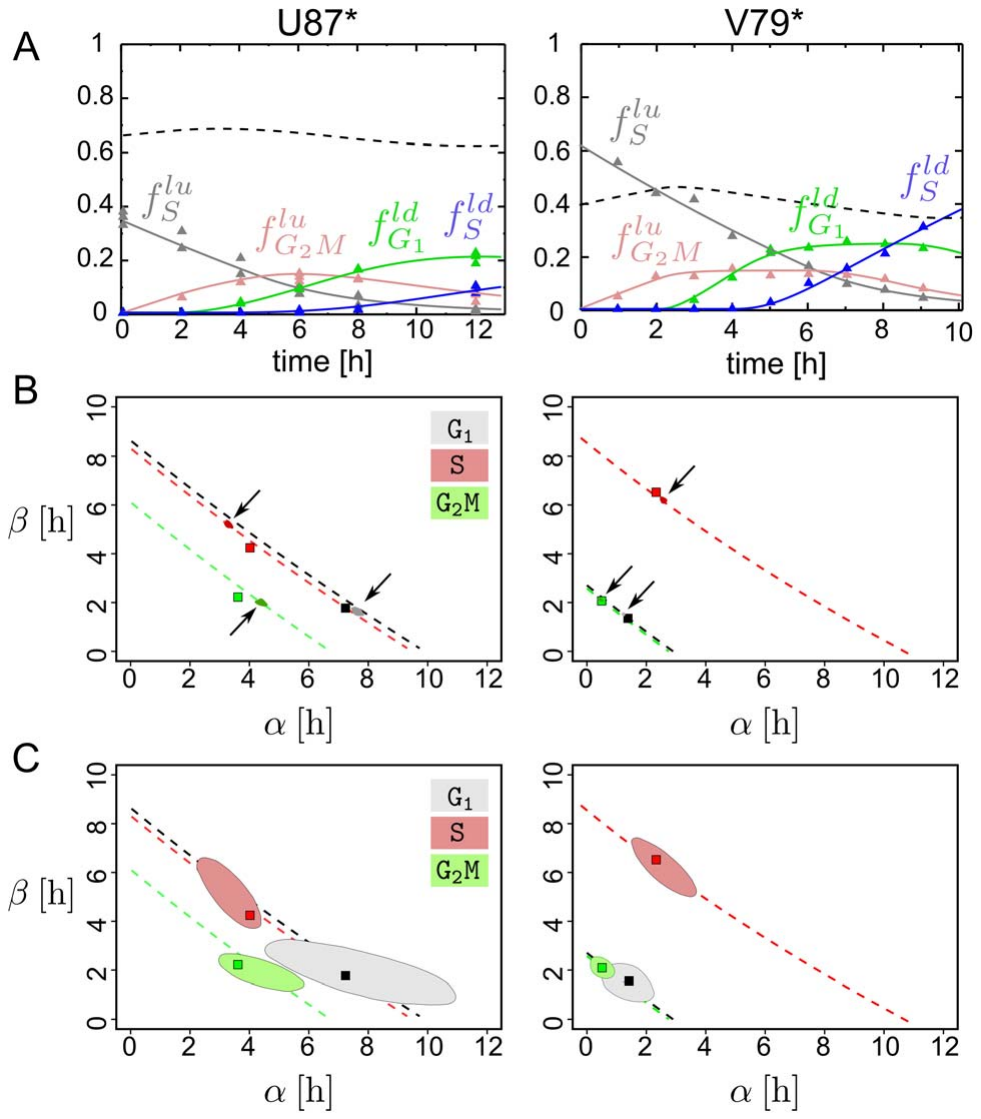
Analysis of simulated dual pulse labelling data. **A**: Average kinetics of unlabelled (dashed line) and labelled cell cohorts (colored lines) were computed from Eq. 25, using ML parameter estimates from the U87 and the V79 data sets (U87: 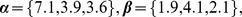 V79: 
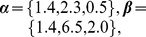
 units are hours). Support points and repeats were chosen according to the real experiments. Multinomial noise was added, mimicking the residuals found in the original data sets (see the Computational Methods section for more details). Finally, model solutions (lines) were fitted to the synthetic data sets (triangles). Best fit parameters (U87: 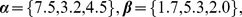 V79: 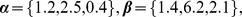 units are hours) **B**: ML parameter estimates from simulated data. All ML regions converge to point estimates (arrows). Squares indicate parameters used for generating the data (see A). **C**: Bayesian bi-variate 99%-credibility regions for the parameters 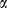 and 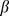 for each phase, based on the artificial data.

To assess if the latter method would allow quantifying more accurately and precisely the parameters of the model, we generated *in silico* data mimicking the output of a hypothetical dual pulse experiment using Eq. 24 and Eq. 25 (see Fig. 5 A). We found that by employing the redesigned protocol with the same replicates and time points as in the corresponding data sets, we could reduce the regions corresponding to the ML up to point estimates (Fig. 5 B). Furthermore, the uncertainties due to noise became also significantly smaller (Fig. 5 C). Pooling this artificial data according to the output expected from a single pulse experiment, reproduced again the uncertainties seen in Fig. 3 C (not shown). Together this indicates that the redesigned dual pulse protocol provides parameter estimates with higher accuracy and precision. Real dual pulse labelling experiments will however be needed to confirm these theoretical predictions.

### Robustness of the estimates to other probability distributions of the phase completion times and to concurrent cell loss

The cell cycle model introduced here is deliberately simple and neglects cell loss. In this section, we ask whether the estimates of its parameters are reasonable when some of the simplifying assumptions of the model do not hold. Specifically, we ask how accurate are the mean and standard deviation of the phase completion times estimated using this simple model if the true completion times were not distributed as a delayed exponential function or if there was concurrent phase-specific cell loss.

**Figure 6:**
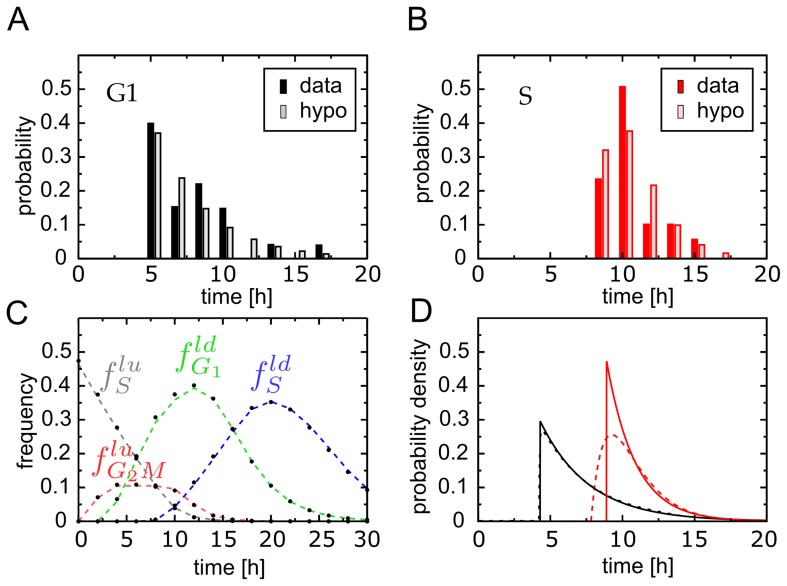
Robustness of parameter estimates to empirical phase duration distributions that are not delayed exponential functions. **A-B**: Least-squares fitting of histograms predicted from a hypoexponential distribution with two decay and one delay parameter 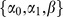 to measurements of phase durations using fluorescent biosensors [35]. The number of cells that were tracked in the original study was around 15 cells. **C**: Best fit of the cell cycle model with delayed exponential completion time distribution densities to synthetic data generated from a model with hypoexponential completion time distribution densities for the 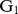 and 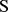 phase with parameters as in A and B. **D**: Recovery of the initial distribution densities (solid lines) using the delayed exponential model (dashed line). Both the average and the variability in the 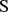 phase completion time distribution (original average: 10.70 h, estimated average: 10.88 h; original std: 2.03 h, estimated std: 1.99 h) were estimated accurately. The data shown in A-B was read from the graphs in the original publication ([35]).

Empirical measurements [35] indicate that the cycle phase time for the S phase is distributed closer to a delayed hypoexponential or a delayed gamma distribution (see below) rather than the caricatural delayed exponential. Therefore, an important question which arises is how much do the estimates of the average and standard deviation in phase durations obtained with this simple model depend on the true underlying distribution? While many different scenarios could be tested we opted to fit a delayed hypoexponential density with two decay and one delay parameter to direct *in vitro* measurements of 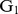 and 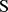 phase durations employing fluorescent biosensors (Fig. 6 A-B, [35]). Using the obtained best-fit estimates, we then performed *in silico* dual-pulse labelling experiments, in which the phase durations were drawn in the case of the 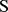 and 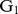 phase from delayed hypoexponential density functions (Fig. 6 C). Finally we fitted the simple model, i.e., Eq. 24 and Eq. 25, which is based on delayed exponential distributions, to this data, to see if we could recover the original averages and standard deviations despite using the ‘wrong’ caricatural model. Both summary statistics (i.e., mean, standard deviation) of phase durations could successfully be re-estimated (Fig. 6 D). Although generalizing this finding lies out of the scope of this article, it suggests that even if the true underlying distribution is not a delayed-exponential function, important quantities like the average and standard deviation of the phase durations may still be estimated with the simple model developed herein. It also indicates that BrdU labelling experiments with a realistic number of samples are unlikely to have the power to discriminate between delayed exponential and more complex density distributions.

We now turn to the issue of how much the presence of phase-specific cell death (or loss in general), which is unaccounted for in our model, affects the accuracy of the estimates of the mean and standard deviation of the phase durations. To this end, we will first introduce the extensions necessary to describe cell death in the model. We rely on the fact that if the probability of death per cell cycle is less that 50%, the average population size will asymptotically grow exponentially with an effective growth rate 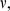 where 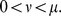 This implies that the arguments used to analyze exponential growth without death remain valid for a model that allows moderate levels of cell death.

To consider death, we assume that cells have two possible fates per phase, either they progress to the next phase or they die. Let 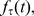 as before, be the phase completion time density, conditioned however on the cell being alive at time 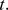 And let 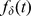 be the phase-specific time to death density conditioned on the cell having not progressed to the next phase. Then, as e.g., in [36], assuming that both events compete with each other (i.e., whatever fate happens first, prevents the other), the resulting density 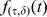 becomes
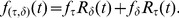
(28)


Consider now a scenario of an exponentially growing population, in which cell death occurs exclusively during phase 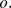 Let us assume further that the 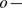
phase specific time to death density 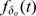 is a simple exponential density with mean 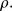 Using straight-forward probabilistic arguments, we can compute analytically, for this simple scenario two important quantities, namely the probability to die in this phase (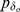), and the expected value of the effective completion time, distributed as 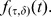 We get
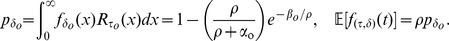



Note that, in this simple case, 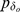 is also the probability to die per division cycle.

**Figure 7:**
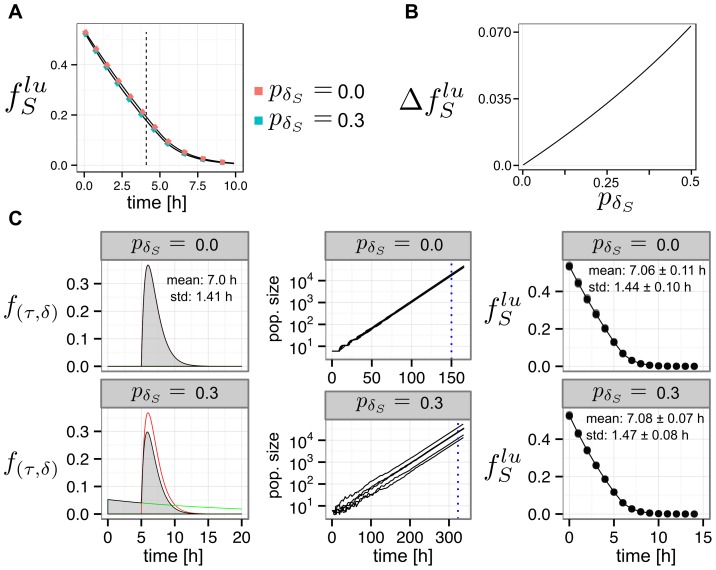
Effect of cell death and completion time distribution on parameter estimates. **A**: Comparison of analytical predictions (lines, Eq. 29) with simulated BrdU labelling experiment (squares). Cell death is assumed to occur exclusively during S phase with probability 0 (red) and 0.3 (blue) respectively. Only the 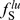 population is considered. Parameters: 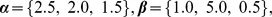 units are hours. **B**: Difference between Eq. 29 (accounting for cell death) and Eq. 24 (neglecting cell death) at time 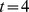 h (see dashed line in A), as a function of 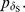 **C**: BrdU labelling experiments were simulated assuming gamma distributed phase completion times (red curve, graphs on left column) and cell death during S phase with probability 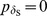 and 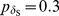 (green curve, graphs on left column). The effective completion time 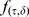 (gray density plot, left column), the population growth (middle column) and the estimation of the mean and the standard deviation of 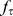 are shown for both cases. Approximate confidence intervals for the estimates are computed as 1.96 times the standard error. Even though 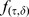 and the population growth are strongly influenced by the value of 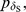 both 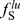 and the estimates extracted from 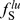 are barely affected. The dashed lines in the middle column indicate the time of the first pulse, which was chosen such that the average population was similar in both scenarios. Parameters for gamma distributed completion time distribution of the three phases: shape: 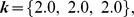 scale: 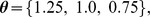 delay: 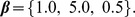

Evaluating Eq. 18 using 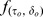 instead of 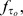 we obtain for Eq. 24,
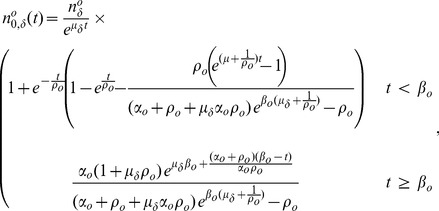
(29)


where 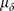 and 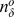 represent the equivalents of 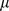 and 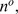 we had previously defined for the case of no cell loss. The former quantities, which now depend on 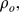 are derived applying to Eq. 5 the same substitution as above. Expressions equivalent to Eq. 10 and Eq. 11 are obtained along the same lines. These become however rather lengthy and are therefore omitted here. Eq. 29 reproduces accurately 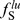 in simulated BrdU pulse labelling experiments, if death occurs, as specified above (see Fig. 7 A for an example with 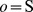 and 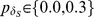). The differences between the analytical predictions for 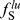 with 30% death and without death (denoted by 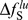) are, for the parameter sets that we tested, relatively small, and vanish as expected, as 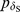 tends to zero (see Fig. 7 B for 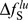 computed at one specific time point (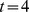 h) for different values of 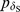).

To further test, how much both cell death and a completion time with a shape distinct from a delayed exponential may jointly affect parameter estimates, we simulated BrdU pulse labelling experiments, where two major assumptions underlying Eq. 24 were simultaneously violated. First, we assumed a delayed gamma distribution (with shape parameter of two) for the completion time of each phase. Second, we considered cell death during 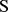 phase, and adjusted 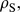 such that 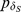 was either zero or 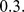 The population size (starting with five cells) took about twice as much time to grow to a similar size for 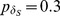 compared to a the scenario without death (see Fig. 7 C, middle column, for five independent simulations). In addition, the variability in the population sizes between the simulations appeared higher for increased death rates. In contrast, when estimating the mean and variance of 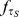 by non-linear least squares fitting using Eq. 24, the marked changes seen in the population kinetics where not paralleled by changes in the estimates. Both the mean and the variance were accurately determined in both cases (see Fig. 7 C, right column). Taken together, this suggests, that the estimates for the mean and variance of 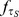 using Eq. 24, at least for the reasonable parameter values that we tested, are relatively robust to simultaneous changes in the shape of the completion time, and moderate levels of cell death.

## Discussion

In this article, we propose a simple stochastic model that aims at approximating the time it takes for a cell to accomplish the sequential phases of the cell cycle, by defining the completion time in each phase as a delayed exponential density distribution. At first sight this might seem a gross oversimplification of all the processes involved. However, when compared with experimental data, this simplistic model performs surprisingly well.

While the observation that the model reproduces closely the experimental time series has to be interpreted with care, we think its success can be explained by the fact that the probability rule captures simultaneously two important regimes of complex biochemical processes that qualitatively differ in their completion time distribution. As was shown recently by Bel *et* al. [37] the completion time for a large class of complex theoretical biochemical systems, including models for DNA synthesis and repair, protein translation and molecular transport, simplify either to deterministic or to exponentially distributed completion times, with a very narrow transition between the two regimes depending on the rate parameters. These are precisely the ‘ingredients’ of the delayed exponential distribution. Under this light our model could be naively interpreted as a sensor that measures approximately the relative contribution of delay and decay processes in each of the cell cycle phases. However, whereas delays connected in series form again a delay, this is not true for decays. Sequentially coupled decays form a process with hypoexponential distributed completion times with a shape similar to the frequency distribution of cell cycle phase completion time reported in [35]. Thus a more flexible model for the completion time of each phase could be a hypoexponential distribution of the family that we are currently using to model the total cell cycle length distribution (i.e., Eq. 3). If instead, processes are not connected in an ordered series but rather concurrent, the times for all the processes to complete is dominated by the largest delay or the smallest decay parameter.

It is tempting to interpret the relative weight of constant delay and exponential decay (i.e., the coefficient of variation) as a measure of the precision of the processes regulating each phase, which in turn might reflect a selective pressure on timing. Tighter pressure might reduce the coefficient of variation, as our results suggest for the 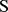 phase when compared to the remaining phases. Yet, this might also reflect the conjunction of many parallel and independent process such as replication forks whose number is expected to increase the timing precision by the law of large numbers. In fact, the mean time and the variance of the 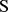 phase are shorter in the early phase of the embryo when cells display a higher number of replication forks in which the DNA polymerase progresses at the same rate [38].

An important simplification of our model consists in the assumption that cell loss by death, differentiation or immigration is small compared to population wide division rates, such that we can neglect it when fitting the model to experimental data. The main reason to adopt this approach was simplicity and the fact that the available data sets did hardly permit the determination of the possibly large number of additional parameters. While for the U87 LIFE/DEAD discrimination was performed, the markers used for gating are specific for late stages of apoptosis or necrosis typically after membrane integrity is lost and therefore do not necessarily reflect the true fraction of dying cells. The fraction of dead cells identified and excluded by this method was typically low. In case that experimental conditions would however suggest substantial cell loss, the model is flexible enough to be adapted without major technical difficulties, along the lines of Eq. 28 and Eq. 29. For instance, when the number of new-born cells equals the number of dying cells, solving the model analytically turns out to be easier, because 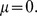 And given that the apoptotic state (e.g., defined by Annexin-V staining) would be measured simultaneously with nucleoside incorporation and DNA content, this could open up the possibility to assess the duration of apoptosis *in vivo*. These potential extensions not withstanding, it is reassuring that considering concurrent phase-specific cell death of up 30% may not change the estimates of the mean and standard deviation of the phase completion time obtained using a caricatural model that neglects cell death, as our results indicate.

Another fundamental abstraction of our model is that the completion times for the cell cycle phases of a given cell are uncorrelated, which also implies uncorrelated division times of parental cells and siblings. Even though positive correlation in division times between parental and daughter cells [10] and between siblings [36] has been observed recently *in vitro* by direct long-term microscopy of activated proliferating B cells, Schultze *et* al. reported many years ago for *in vivo* murine crypt epithelial cells the lack of correlation of completion times of a cell through successive phases [31]. It remains to be shown experimentally how much of the correlation or lack of correlation is due to cell type or environment. In any case, it would be interesting to extend the present model to include correlation in phase completion times.

The live cell biosensor-based fluorescent imaging strategy exploited in [35] allows for direct quantification of the stochastic timing of the cell cycle phases. It is worth comparing the estimates of cell cycle phase-specific completion times obtained with this direct method with those provided by the indirect pulse labelling method. The mean 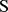 phase completion time was reported for the lines NCI-H292 and HeLa cell line to be 8.2 and 8.4 hours respectively with standard deviation of 0.5 and 2.9 hours (extracted from Fig.2 in [35]), which lie in the range of the estimates we obtained, despite the different human cell lines that have been analyzed (Table 1). In principle, pulse labelling with nucleoside analogs can be used *in vivo* to quantify the stochasticity of the cell cycle in anatomical places that are currently not feasible to visualize by multiphoton microscopy, given that a sufficiently large (over 1000) and representative sample of cells can be harvested. Our method therefore provides, concerning the 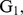 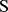 and 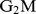 phases, very similar information as these imaging methods, yet it has a much wider application scope.

In comparison with the Smith and Martin cell cycle model, that assumes a single variable phase [5], we have proposed a more complex model with three variable phases. A question can be raised whether a less complex model with variability in only one or two of the three phases would reproduce equally well our BrdU pulse labelling data. This could simplify the analysis and reduce the issue of parameter identification. One might, for example, consider a scenario, similar to the double transition probability model analyzed in [39], in which the 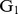 and the 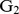 phase have delayed exponentially distributed durations, while the durations of 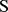 and 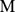 phase are fixed. It is easy to see that such a less complex model is embedded into our model, as it suffices to set 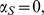 while assuming that the variability in the duration of the 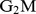 phase is generated entirely during the 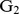 phase. Clearly, from a data fitting perspective, and especially for the V79 data set, the simpler embedded model and the larger model would perform equally well. This can be read directly from Fig. 3, as the set of approximate ML estimates for 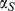 includes values that are equal or close to zero. However, the interpretation of the V79 data set based on these two models would be fundamentally different. For instance, by relying on the deterministic model, one would be lead to conclude that the 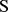 phase duration is for every cell about 9 hours. By allowing however for 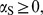 possible interpretations of the data encompass the latter case, but in addition include scenarios in which some cells complete their 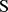 phase in about 7 hours, while other cells may take far longer. Even though the original data does not permit to discriminate between these models, simulated dual pulse labelling experiments indicate that this is in principle feasible. Finally, in view of the experimental data provided by Hahn *et* al. ([35], used in Fig. 6 B), the scenario of variable S-phase duration with 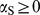 is well justified.

On the other hand, we distinguished only three cell cycle phases, although the cell cycle is typically structured into at least four biologically distinct phases. This simplification stems from the fact that quantification of DNA content by flow cytometry cannot discriminate between cells in the 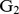 and 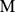 phase. Additional biomarkers, such as pS780 reported by Jaccoberger *et* al [40], could be used together with DNA content dyes and nucleoside analogs in extended labelling protocols to identify the four main cell cycle phases. Extending the model to distinguish accordingly a fourth phase would be rather straightforward mathematically. Despite restricting the model to three phases, it is worth noticing that we are extending the work of Cain and Chau [39], [41], who studied both balanced and non-balanced growth conditions, assuming one and two random transitions, mapped respectively to part of 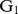 and the remaining cell cycle phases. Also, we extend the work of Larsson *et* al. [42] who were able to infer the variation in the completion times of 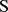 and 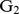 based on the histograms of DNA content.

Long-term labelling with BrdU has been used *in vivo* to study disease progression of infected rhesus macaques with the simian immunodeficiency virus [1], [43] and due to toxicity more rarely in HIV-1 [44]. These studies typically targeted turnover rates of T lymphocytes subpopulation over a time period of several weeks and provided average birth and death rate estimates. In contrast, the method outlined here measures cell proliferation at a much short timescale 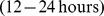 and has the potential to yield phase specific estimates of both the average and the variability of completion times. We anticipate that valuable complementary information about SIV and HIV infection could be gained using the redesigned protocol proposed here, especially in the light of the known modulation of the cell cycle checkpoints by accessory viral proteins [45].

Recently, in a computational ‘tour de force’, Falcetta et al. [25] used a stochastic model of cell cycle progression with discrete age-structure to derive qualitative conclusions about the mechanism of action of several anti-cancer therapies. This model was able to mimic (in their wording ‘rendering’) quantitative data on single BrdU pulse labelling assay and time-lapse imaging. The empirical distribution cell cycle lengths they reported is akin to the hypoexponential family in our model, however, the distributions of phase lengths remain implicit in their simulation framework, in which time is discrete and the parameters are transition probabilities per time step. This prevents knowing how uncertain are the estimates of the phase length variances based on single pulse labelling using their approach.

Dual pulse labelling with a pair of thymidine analogs has been used before to study cell cycle kinetics [19], [28], [31]. What is common to those studies is scheduling the two consecutive pulses by fixing the time lapse between the pulses, irrespectively of the time at which cell samples are collected for cell cycle phase analysis. It is worth stressing that, according to the present study, specially when the second pulse is timed according to each individual sample (i.e. adjusting accordingly the interval between pulses) one can harness the potential of the model to quantify the mean and variance of the phase-specific time. Making the second pulse at a fixed minimal time before collecting cells for analysis allows to resolve cellular cohorts, which would otherwise be confounded.

New technologies like the one developed by Hahn et al. [35] but also the ubiquitination-based cell cycle indicator, termed ‘Fucci’ [46] will greatly increase our understanding of phase resolved cell cycle progression and unveil its epigenetic and stochastic variability in isogenic cell populations. To translate this knowledge gained mainly from *in vitro* cell cultures into an *in vivo* context, long term (greater than 12 hours) and continuous multi-photon imaging may be required. This however is technically very demanding, and may remain prohibitive for cells deep inside tissues despite major technological advances in the field. The methodology presented here allows to measure phase specific cell cycle progression variability *in vivo* by relatively simple technical means. Even though nucleoside analogs are potentially carcinogenic, the adverse effects of low dose pulse labelling remain typically undetectable. Determining accurately cell cycle progression variability in mouse models of cancer might become a crucial step in understanding the high variability in susceptibility to cell cycle specific anti-cancer drugs.

## Materials and Methods

### Stability analysis

Here we will show that a cell population that follows the stochastic model specified before will eventually enter a stationary exponential growth phase. The requirement for such an asymptotic behavior is, recalling Eq. 11, that the complex valued function
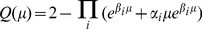
(30)


has for positive valued elements of the vectors 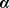 and 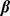 a unique positive real root which represents the upper bound of the real part of any of its other potentially infinite number of roots. The complex numbers 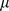 that solve Eq. 30 correspond, according to our model, to the stationary phase growth rate of the proliferating cell population. In case that 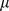 is real, the population is growing exponentially, while if 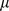 is purely imaginary growth is oscillating. In general, roots have both non-zero real and imaginary parts, which leads to oscillations with growing or decaying amplitude. If for real 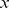 and 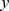 we write 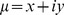 the real and imaginary part of 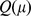 are computed as
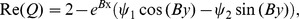


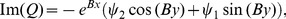
(31)


where
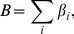


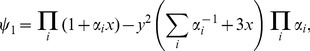
(32)

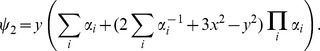



For 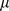 to be a root of 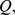 both real and imaginary part have to vanish.We restrict our analysis to the positive complex half plane, i.e. 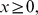 since we are interested in growing and not contracting cell populations. Due to the symmetries in the trigonometric functions 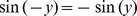 and 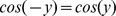 and 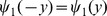 and 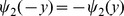 one can easily see that if 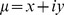 is a root, its complement 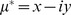 is also a root. We can thus reduce the analysis even further to values with positive imaginary parts. If for fixed 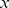 we plot 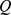 in the complex plane as a parametric function of 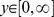 we get a spiral with the distance from a center point 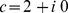 given by
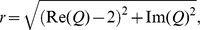


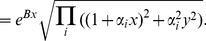
(33)


**Figure 8:**
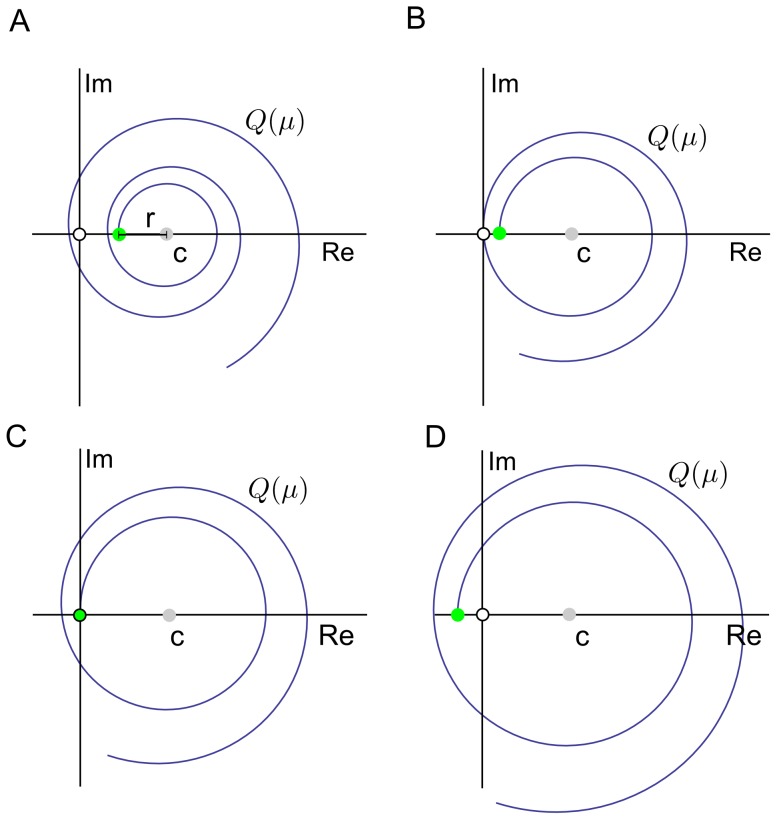
Stability analysis. 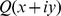
 as a function of 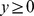 for fixed values of 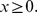 For 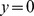 (green circle) the real part of Q takes, depending on 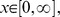 a value in the interval 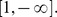 The values for x are increasing from A-D, while 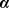 and 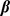 remain unchanged. For relatively low values of 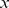 (A-B) the real part 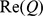 is positive for 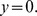 After one or several turns, i.e by increasing 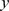 the spiral can potentially cross the origin only once (empty circle). In A the spiral misses the origin, while in B the spiral crosses the origin after one turn. Crossing of the origin means that the corresponding complex number 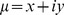 is a root of Q. In C the spiral starts at the origin. This represents the only real positive root of Q. For initially negative values of 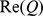 (D) the spiral can never cross the origin because the distance to the center point (gray circle) is already in the beginning for 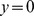 larger than the distance between the latter and the origin. By increasing y this distance will even grow further according to Eq. 33.

Crucially, as 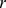 is a monotone increasing function of 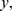 the spiral never crosses itself. For 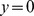 the imaginary part of 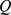 vanishes as expected because 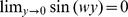 and 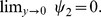 For this special case 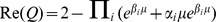 is obviously monotone decreasing with 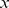 and restricted to the interval 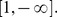 This means that the spiral can only ‘start’ in the interval between one and minus infinity. Taken together, this implies that if for 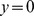 and fixed 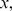 the real part of 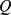 is positive, then there exist a single ‘opportunity’ to cross the origin, while if negative there exists none. At the border where the real part is zero (Fig. 8 C), the corresponding value of 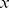 is the only positive real root. Due to the monotonicity of 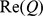 any value of 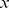 greater than the positive real root will result for 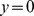 in 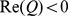 which does not admit for any solution. The different possible scenarios are exemplified in Fig. 8.

### Experimental methods

#### Cell culture

Human astrocytoma cells U-87 MG (ATCC-LGC) were routinely cultured with Dulbecco's modified Eagles medium (DMEM, Biochrom AG) supplemented with non-essential amino acids (NEAA, Invitrogen GmbH), heat-inactivated fetal bovine serum (FBS, 10%, Biochrom AG) and additives (penicillin-streptomycin-glutamine, Invitrogen GmbH) in plastic flasks (TPP AG) at 37°C in 5% CO_2_-humified incubators and were passaged twice a week using Dulbecco's PBS (DPBS, Apotheke Innenstadt Uni Mnchen) and Trypsin/EDTA (Biochrom AG) before reaching confluence.

#### Treatment with BrdU

For cell cycle analysis cells (2.0×10^4^ cm^−2^) were seeded in 75 cm^2^ culture flasks and incubated for 24 h followed by the BrdU pulse. For this purpose, medium was replaced by medium supplemented with BrdU (10 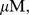 Bromodeoxyuridine, Becton Dickinson GmbH), cells were incubated for 30 min at 37°C followed by washing away of BrdU for two times with fresh medium. Cells were then again incubated at 37°C for a designated period of time (0 h, 2 h, 4 h, 6 h, 8 h, 12 h) to measure proliferation over 12 h.

#### Preparation of samples

Collecting of cells was performed by trypsinization using DPBS, Trypsin/EDTA and medium followed by washing of cells in DPBS. To exclude dead cells from the analysis staining of dead cells was performed. For this purpose cells were incubated for 30 min with fluorescent dye (LIVE/DEAD Fixable Green Dead Cell Stain Kit, Invitrogen) according to the manufacturers instructions followed by washing with DPBS. Consequent steps of sample preparation were processed using the APC BrdU Flow Kit (Becton Dickinson GmbH). Cells were washed once with Perm/Wash Buffer and fixed for 30 min on ice with Cytofix/Cytoperm Buffer. After washing with BD Perm/Wash Buffer cells were resuspended in Cytoperm Plus Buffer and incubated on ice for 10 min followed by washing with Perm/Wash Buffer and incubation in Cytofix/Cytoperm Buffer for 5 min on ice. Cells were then washed with Perm/Wash Buffer and incubated with 2 M HCl-Triton (1%) for 30 min at room temperature followed by washing twice with Perm/Wash Buffer. For detection of incorporated BrdU cells were incubated with diluted (1∶50) fluorochrome-conjugated anti-BrdU antibody for 20 min at room temperature. Cells were then washed with BD Perm/Wash Buffer and further incubated with DAPI (0.5 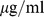 in staining buffer: 100 mM Tris, pH 7.4, 150 mM NaCl, 1 mM CaCl_2_ 0.5 mM MgCl_2_ 0.1% Nonidet P-40) for 30 min at room temperature. All samples have subsequently been stored on ice until acquisition.

#### Acquisition and analysis

Acquisition of data was performed by measuring fluorescence intensity using a BD LSR II Cytometer at the excitation wavelength of 660 nm for APC and 450 nm for DAPI and the software BD FACSDiva.

### Computational methods

#### Modeling and simulations

Anti-derivatives, equations as well as eigenvalue problems were solved with the help of Mathematica. Stochastic simulations, Markov chain Monte Carlo and optimization algorithms (e.g. least square fitting and Newton-Raphson root finding) were implemented in C++. To fit the parameters of the model to the data we relied on the population based covariance matrix adaptation evolution strategy provided by the C++ library SHARK [47].

#### *In silico* data

In order to anticipate and compare the information content in data sets that could potentially be acquired according to the dual-pulse protocol, *in silico* data was generated. The simulated data consisted of frequencies computed according to our model using ML parameter estimates. Noise was added to the frequencies by simulating a sampling process with replacement with frequencies given by the model and a population size of 300 and 600 for the U87 and the V79 data set respectively. This reproduced approximately the variability observed in the original data sets. To make comparison with available data reasonable support points were taken to be the same as in the respective data set.

#### Bayesian inference

When estimating, by FACS analysis, frequencies of cells in different phases of the cell cycle, measurement noise becomes unavoidable. Potential sources of noise include variability in experimental conditions, gating errors, stochasticity in cell division, FACS measurement errors, and many more. Here we describe an attempt to account, in a simple way, for the observed experimental noise by taking a Bayesian approach. This provides us not only with maximum likelihood estimate regions of the model parameter, but in addition will give us an idea about the uncertainty that we have about the parameter values.

Even though considering all potential sources of noise would be most consistent, the resulting probability model would become far more complex than our initial cell cycle model. To avoid this overload we assume that a relatively simple *ad hoc* multivariate probability density function approximates reasonably well the average and the noise in the observed frequencies at a single time point. This probability density function, which corresponds to the likelihood 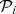 of a single measurement event 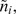 is defined by
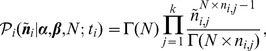
(34)


where 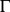 is the Euler gamma function. The right-hand side of Eq. 34 corresponds to a continuous approximation of a scaled multinomial distribution with support 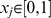 and 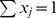 [48]. The parameter 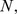 which determines the spread of the distribution, can be interpreted as an effective population size. Taking e.g., a sample of size 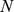 from a population of cells containing 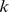 sub-populations with proportions given by 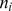 yields frequencies with a probability density approximately distributed accordingly. If 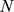 is small the density distribution is broad, while if 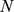 becomes large the density distribution becomes narrow.

Following in general terms the notation in the main text, the 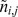 denote the 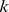 measured population frequencies from experiment 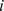 and the 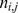 stand for the corresponding frequencies predicted by the cell cycle model. The latter obviously depend on the parameter vector 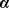 and 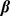 and the time 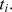


Having defined the likelihood 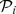 for an outcome of a single pulse labelling experiment, the likelihood for the outcomes of a set of 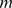 experiments is the product 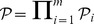 under the reasonable assumption that noise in a specific experiment is independent of all the other experiments. By numerically inverting 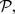 using Bayes theorem, one can obtain the posterior and subsequently the uncertainty over the model parameter given the data, the model and prior knowledge.

To estimate the maximum likelihood regions, the posteriors and the uncertainties in the 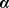 and 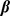 for the U87 and V79 data sets, we implemented in C++ the adaptive Markov-Chain-Monte-Carlo algorithm proposed in [49]. The estimates for the maximum likelihood regions are obtained by fixing 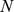 to a very large value (e.g., 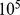). For Bayesian inference, 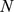 was considered as an additional parameter. For simplicity, improper priors uniformly distributed over the positive real number were assumed for all parameter. The first 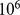 steps of the initially 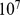 step-long chains were discarded, and of the remaining chains every 1000′th step was included in the subsequent analysis. The credibility regions were computed from the resulting MCMC chains using the ‘HPDregionplot’ routine in the R package ‘emdbook’, and convergence of the chains were confirmed using the Gelman convergence test.
